# Cation–π interactions in competition with cation microhydration: a theoretical study of alkali metal cation–pyrene complexes

**DOI:** 10.1007/s00894-017-3302-3

**Published:** 2017-03-23

**Authors:** Hasan Pašalić, Adelia J. A. Aquino, Daniel Tunega, Georg Haberhauer, Martin H. Gerzabek, Hans Lischka

**Affiliations:** 1 Institute for Theoretical Chemistry, University of Vienna, Währinger Strasse 17, 1090 Vienna, Austria; 2 School of Pharmaceutical Sciences and Technology, Tianjin University, 92 Weijin Road, Nankai District, Tianjin, 300072 People’s Republic of China; 3 Institute for Soil Research, University of Natural Resources and Life Sciences Vienna, Peter-Jordan-Strasse 82, 1190 Vienna, Austria

**Keywords:** Cation–π interactions, Microhydration, Alkali metal cation–pyrene, DFT

## Abstract

Cation–π interactions were systematically investigated for the adsorption of H^+^ and alkali metal cations M^+^ to pyrene by means of Møller–Plesset perturbation theory (MP2) and density functional theory (DFT). The main aims were to determine the preferred adsorption sites and how the microhydration shell influences the adsorption process. The preferred adsorption sites were characterized in terms of structural parameters and energetic stability. Stability analysis of the M^+^–pyrene complexes revealed that the binding strength and the barrier to transitions between neighboring sites generally decreased with increasing cation size from Li^+^ to Cs^+^. Such transitions were practically barrierless (<<1 kcal/mol) for the large Rb^+^ and Cs^+^ ions. Further, the influence of the first hydration shell on the adsorption behavior was investigated for Li^+^ and K^+^ as representatives of small and large (alkali metal) cations, respectively. While the isolated complexes possessed only one minimum, two minima—corresponding to an inner and an outer complex—were observed for microhydrated complexes. The small Li^+^ ion formed a stable hydration shell and preferentially interacted with water rather than pyrene. In contrast, K^+^ favored cation–π over cation–water interactions. It was found that the mechanism for complex formation depends on the balance between cation–π interactions, cation–water complexation, and the hydrogen bonding of water to the π-system.

## Introduction

Cation–π interactions are among the strongest noncovalent interactions; in many cases they are stronger than hydrogen bonds and van der Waals interactions [1, 2]. They are frequently encountered in nature and play pivotal roles in complex biological systems such as cation complexes with proteins, chemical catalysis, and solid-state physics [1, 3, 4]. Another interesting area where cation–π interactions play a significant role is soil chemistry. Cation–π interactions occur in several adsorption processes where metal cations interact with aromatic moieties in soil organic matter and black carbon [2]. These interactions have been considered to be more important than π–π stacking and hydrophobic interactions [2]. The nature of cation–π interactions is not purely electrostatic. A symmetry-adapted perturbation theory (SAPT) study by Singh et al. [5] demonstrated that, besides the electrostatic component, interactions due to induction and dispersion make an important contribution. Cation–π interactions have been studied theoretically for various π-systems, predominantly with mono- and divalent cations, using a wide variety of methods ranging from density functional theory to high-level ab initio methods. Typical cations studied in this context are the alkali and alkali earth metal cations, while benzene is the typical representative of π-systems [6–24]. Theoretical studies have also investigated transition metal cations such as Zn^2+^ and Cd^2+^ [25]. Several experimental investigations performed in the gas phase using mass spectrometric methods have been carried out to study cation–π interactions, with benzene employed as the aromatic system [1, 2, 26]. For example, Sunner et al. [27] reported in 1981 that the K^+^–benzene interaction is stronger than the K^+^–water interaction. About 20 years later, Cabarcos et al. [28, 29] found that K^+^ favors cation–π (benzene) over cation–water interactions using infrared spectroscopic and mass spectrometric methods. In stark contrast, Na^+^ was found to prefer the aqueous environment. These findings were crucial to advancing our understanding of selective ionic channels, as hydrated Na^+^ is too large to pass through a pore. In addition to theoretical studies of small molecules such as benzene, attention has also been directed towards larger aromatic systems. For example, Burk et al. [30] studied the interactions of Cs^+^ with a set of neutral and anionic compounds related to soil organic matter (e.g., unsubstituted and substituted aromatic compounds such as phenolic acids and phenolates) theoretically. The authors found that this cation had greater affinity for the substituted aromatic compounds than the unsubstituted ones. Gal et al. [31] investigated the adsorption of Li^+^ to large polycyclic aromatic hydrocarbons (PAH) up to circumcoronene. They showed that the interaction energy decreases with increasing number of fused rings. Similar trends were reported for the transition state barriers in that work. Various linear and branched PAHs were chosen to represent graphene nanoflakes, and their interactions with Li^+^ and Mg^2+^ ions were studied theoretically, which highlighted an effect of ring size on the stability of the cation–π complexes [12]. Studies of cation–π interactions of alkali and alkaline earth cations with graphene models using density functional theory (DFT) with the M06-2X functional [32] showed that the binding strength depends significantly on the cation studied [33]. The geometries, electronic structures, and magnetic properties of alkali earth metal atoms absorbed onto graphene have also been investigated by DFT calculations using periodic boundary conditions [34].

As previously noted, cation–π and cation–water interactions generally compete. Thus, it is important to consider solvent effects, especially explicit solvent water molecules, in simulations. In a combined experimental/computational study, Meng et al. [35] investigated the adsorption of Na^+^ and K^+^ to a graphite surface, including hydration effects. Their results were in general agreement with those obtained by Cabarcos et al. [29] in a previous experimental work on Na^+^–benzene and K^+^–benzene complexes.

In the work reported in the present paper, in order to improve our knowledge of the characteristics of the adsorption of alkali cations to larger PAHs, the processes associated with the adsorption of a whole series of alkali metal cations (Li^+^ to Cs^+^) to pyrene were studied systematically. In contrast to benzene, pyrene is large enough to represent π-systems that possess several potential adsorption sites, but is also sufficiently small to allow us to perform extensive quantum chemical calculations on it. It can be further used as a prototype model for nanographene flakes or, more generally, as an initial model for the surface of black carbon, since graphitic layers are a major component of natural black carbon [36]. Pyrene and pyrene-carbon vacancy defect structures have also been successfully used to study the chemisorption of a hydrogen atom onto graphene nanoflakes [37]. The present study of the interactions of alkali cations was completed by exploring the bonding of H^+^ to pyrene. This work particularly focused on attempting to understand microhydration effects and the aforementioned competition between hydration and cation–π interactions. Thus, the role of the first hydration shell in adsorption structures is discussed here in detail, paying special attention to hydrogen bonding and the balance between hydration and direct adsorption.

## Computational methods

Cation–π interactions were investigated for M^+^–pyrene complexes (M = H, Li, Na, K, Rb, and Cs) by means of second-order Møller–Plesset perturbation (MP2) theory [38, 39] and density functional theory (DFT) using the exchange-correlation functional PBE [40, 41]. For both methods, the resolution of the identity (RI) approximation [42, 43] and a flexible def2-TZVPP basis set were applied [44]. The computationally efficient RI approximation has been demonstrated to give almost identical results for interaction energies and equilibrium distances of cation–π complexes to those afforded by pure MP2 and DFT methods [45]. The def2-TZVPP basis set with relativistic effective core potentials (ECP) was used for rubidium and cesium [46, 47]. For all of the complexes studied, full geometry optimizations were performed in the gas phase at the MP2 and PBE levels. Transition states connecting the located energy minima for the M^+^–pyrene complexes were optimized by means of PBE. The nature of each structure obtained was characterized by a vibrational mode analysis. Harmonic vibrational frequencies and thermodynamic properties were calculated within the standard harmonic oscillator—rigid rotator—ideal gas approximation for* T* = 298 K and* p *= 1 atm. The final gas-phase interaction energies of the M^+^–pyrene complexes were corrected for basis set superposition error (BSSE) using the counterpoise method as proposed by Boys and Bernardi [48]. Potential energy curves for the approach of isolated and hydrated Li^+^ and K^+^ ions to the pyrene surface were calculated at the PBE level, considering the first hydration shell. Li^+^ and K^+^ were selected as representatives of small and large alkali metal cations, respectively, with the first hydration shells comprising four (Li^+^) and eight (K^+^) water molecules, respectively. Throughout those calculations, the water molecules were fully optimized for each fixed M^+^···pyrene distance. All calculations were performed using the Turbomole program [49].

## Results and discussion

### H^+^–pyrene

**Fig. 1:**
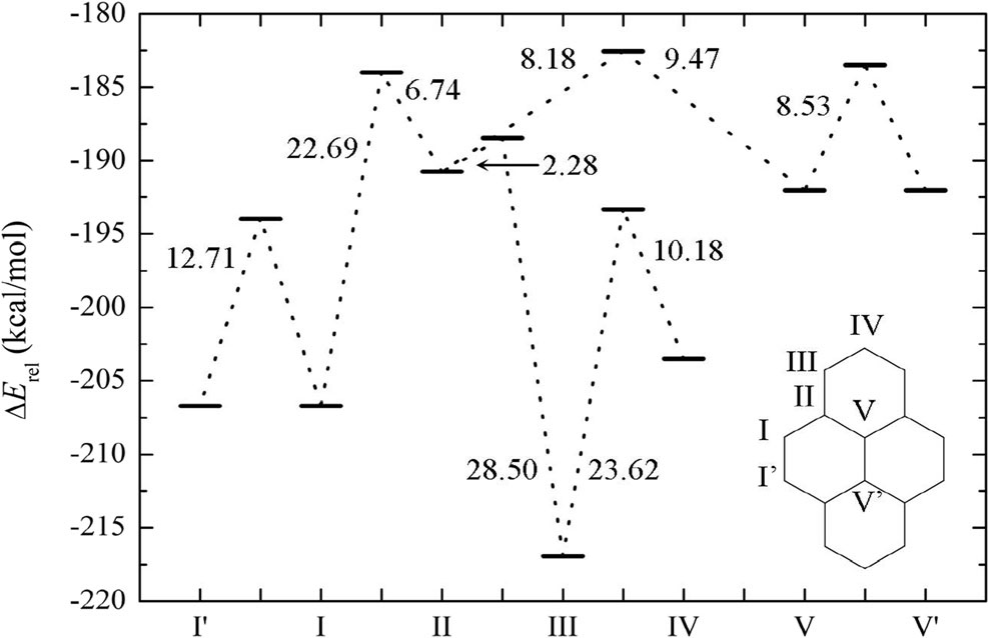
Energy profile and transition barriers (kcal/mol) for H^+^–pyrene, calculated using the PBE/def2-TZVPP method. Sites I and I′ are equivalent due to symmetry, as are V and V′

The stability of protonated pyrene with respect to protonation sites I–V (Fig. 1; sites I–V are peripheral carbon atoms of pyrene as well as an internal carbon) was studied by means of MP2 and PBE calculations. The interaction (formation) energies Δ*E*_f_, the enthalpies Δ*H*_f_, the Gibbs free energies Δ*G*_f_, and the relative energetic stabilities ΔΔ*E*_r_ are collected in Table 1 for both methods. The following order of stability is observed in the results from both MP2 and PBE: III < I < IV < V < II. Protonation of the outer carbon atoms, leading to the formation of C–H bonds, is preferred (III, I, and IV). During the protonation of these sites, H^+^ is bound covalently to the carbon atom, resulting in a CH_2_ group, and a positive charge is generated on the neighboring carbon atom. The resulting C–H bond in the H^+^–benzene system has previously been shown to be covalent [50], and is in good agreement with our result. Site III (the α or C1 carbon of pyrene) is the most stable of the three peripheral carbon atoms, with calculated relative MP2 energy differences (ΔΔ*E*_r_) with respect to sites I and IV of 10.73 and 15.77 kcal/mol, respectively. This is in good agreement with results obtained in earlier computational investigations, which afforded values of 9.9 and 15.7 kcal/mol (HF/6-31G) [51] and 10.5 and 15.0 kcal/mol (B3LYP/6-31G) using significantly smaller basis sets [52]. Preferential proton attack on the α-carbon was also observed in an NMR study by Laali [53] and was qualitatively explained by the larger number of resonance structures in this case compared to the other protonated pyrene structures [51].

**Table 1:** Formation energies Δ*E*_f_, energetic stabilities ΔΔ*E*_r_ with respect to site III, enthalpies Δ*H*_f_, and Gibbs free energies Δ*G*_f_ at *T* = 298 K for energy minima I–V (see Fig. 1) of the H^+^–pyrene system calculated at the RI-MP2 and RI-PBE levels using the def2-TZVPP basis set. All values are given in kcal/mol

H^+^	Δ*E*_f_	ΔΔ*E*_r_	Δ*H*_f_	Δ*G*_f_
RI-MP2				
I	−196.83	10.73	−191.22	−184.90
II	−180.64	26.92	−175.74	−169.37
III	−207.56	0.00	−201.61	−195.25
IV	−191.79	15.77	−186.35	−179.98
V	−180.71	26.85	−175.89	−169.44
RI-PBE				
I	−206.69	10.27	−201.16	−195.01
II	−190.74	26.22	−185.86	−179.46
III	−216.96	0.00	−211.05	−204.78
IV	−203.51	13.45	−198.29	−192.06
V	−192.03	24.93	−186.98	−180.49

The protonation of pyrene at carbon atoms that are not already bonded to H atoms is energetically considerably less favorable. Similar MP2 interaction energies (differing by only 0.1 kcal/mol; Table 1) are computed for site V (a central carbon atom; see Fig. 1) and site II. The relative energy difference from the most stable structure (III) amounts to about 27 kcal/mol. The order of stability found for the interaction energies is preserved for the enthalpies and Gibbs free energies (Table 1).

The interaction energies calculated at the PBE level are stronger by about 10 kcal/mol than those obtained using MP2, whereas the relative energies ΔΔ*E*_r_ yielded by the two methods are in good agreement (differing by <2.5 kcal/mol; Table 1). Since PBE reproduces the order of stability and the relative energy differences afforded by MP2, it was further used to calculate the transition barriers. The energy barriers (kcal/mol) to transitions between neighboring protonation sites are presented in Fig. 1. As can be seen, only the transition from the least favorable structure (II) to the most stable one (III) has a relatively small energy barrier (2.3 kcal/mol). All the other barriers are close to or greater than 10 kcal/mol.

### Alkali metal cation–pyrene

**Fig. 2:**
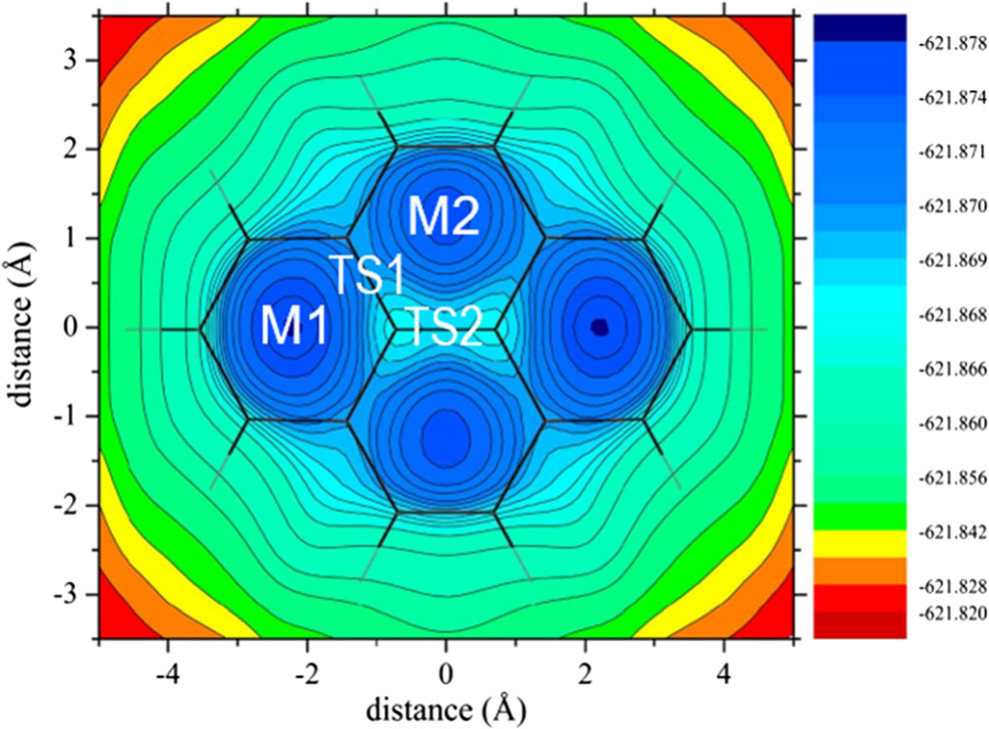
PBE/SVP-calculated energy contour map for the Li^+^–pyrene complex with a Li^+^···pyrene distance of 1.8 Å. Potential energy minima and transition states are indicated in the figure

**Fig. 3:**
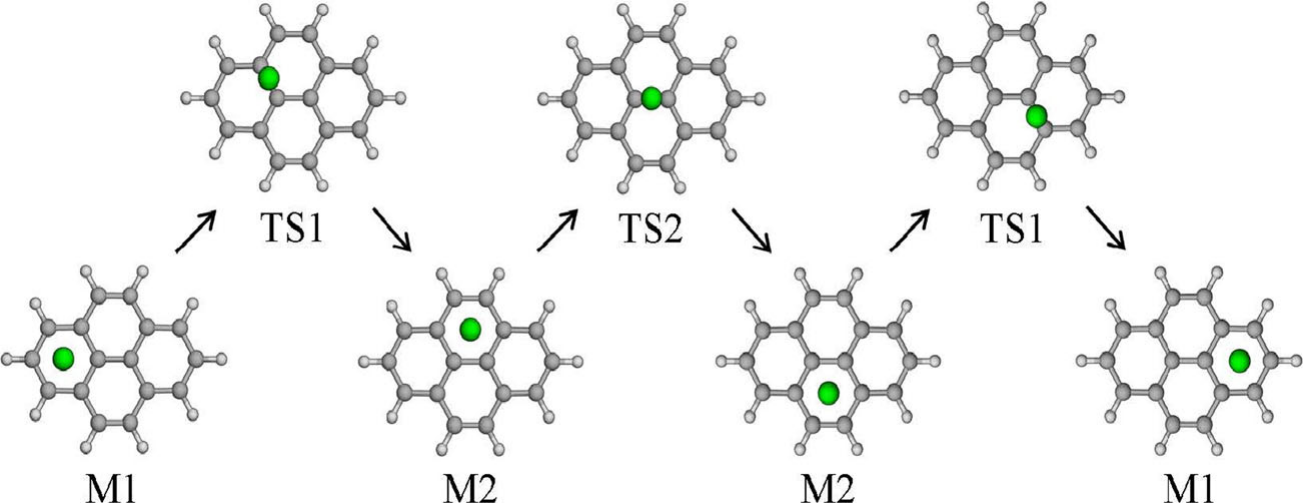
Minimum-energy sites (M1 and M2) and transition states (TS1 and TS2) for alkali metal cation–pyrene complexes

The stabilities of the alkali metal cation–pyrene complexes were investigated by means of MP2 and PBE calculations. To localize the energy minima and transition states of the alkali metal cation–pyrene complexes, the pyrene surface was initially scanned by moving the Li^+^ cation over the pyrene plane at a fixed distance of 1.8 Å from it using the PBE functional and the SVP basis set. The pyrene geometry was kept fixed. From the resulting energy contour plot (Fig. 2), two potential energy minima (M1 and M2) and two potential transition states (TS1 and TS2) were localized (Fig. 3). In the subsequent calculations, alkali cations were placed at the M1/M2 sites and their final positions were then optimized. Similarly, for the transition states, the cations were placed in the TS1/TS2 positions and their positions perpendicular to the pyrene plane were optimized.

M1 corresponds to the structure where the alkali metal cation is located above the outer pyrene ring (Fig. 3). In the case of M2, the alkali metal cation is located above the inner pyrene ring. The MP2- and PBE-calculated interaction (formation) energies (Δ*E*_f_), enthalpies (Δ*H*_f_), and Gibbs free energies (Δ*G*_f_) for both energy minima are collected in Table 2.

**Table 2:** BSSE-corrected formation energies Δ*E*_f_, enthalpies Δ*H*_f_, and Gibbs free energies Δ*G*_f_ at *T* = 298 K for the energy minima (M1, M2) of alkali metal cation–pyrene complexes, as calculated at the RI-MP2 and RI-PBE levels using the def2-TZVPP basis set. All values are given in kcal/mol

	Δ*E*_f_	Δ*H*_f_	Δ*G*_f_	Δ*E*_f_	Δ*H*_f_	Δ*G*_f_
M1	RI-MP2			RI-PBE		
Li^+^	−41.90	−40.97	−33.83	−45.49	−44.52	−37.34
Na^+^	−27.11	−26.45	−19.90	−29.11	−28.89	−22.71
K^+^	−21.63	−21.01	−14.66	−20.36	−20.13	−14.52
Rb^+^	−20.84	−20.16	−13.64	−18.29	−17.70	−12.02
Cs^+^	−21.32	−20.63	−14.20	−17.03	−16.48	−10.85
M2	RI-MP2			RI-PBE		
Li^+^	−40.46	−39.67	−32.71	−43.37	−42.48	−35.44
Na^+^	−26.74	−26.13	−19.73	−28.23	−27.99	−22.02
K^+^	−21.64	−21.03	−14.72	−19.94	−19.63	−14.05
Rb^+^	−20.90	−20.23	−13.66	−17.98	−17.36	−11.82
Cs^+^	−21.49	−20.82	−14.21	−16.72	−16.13	−10.67

Inspection of the MP2 results (Table 2) indicates that the interaction energy decreases with increasing alkali metal cation size except that it is less for Rb^+^ than for Cs^+^ (although the difference between those two values is a few tenths of a kcal/mol). It is also apparent that the MP2-calculated Δ*E*_f_ values for the larger cations K^+^, Rb^+^, and Cs^+^ lie within 1 kcal/mol of each other. The order of stability is the same for both M1 and M2 (Table 2). For Li^+^, the outer pyrene ring (M1, Fig. 3) is the preferred adsorption site; the difference in energy from the M2 site amounts to about ∼1.4 kcal/mol. This difference decreases to ∼0.4 kcal/mol for Na^+^. For the larger cations it is almost negligible, indicating that adsorption to the different pyrene rings is equally probable for the larger alkali metal cations (Table 2). The preference of the smaller cations Li^+^ and Na^+^ for the M1 adsorption site can be explained by the molecular electrostatic potential of pyrene, which is more attractive above the outer (M1) than the inner (M2) ring, respectively [31]. When the interaction energies (Δ*E*_f_, Table 2) are calculated at the PBE level, the trend observed for the MP2 results is basically preserved. The PBE binding energies also decrease steadily with increasing cation size. This trend was also reported for the stability of M^+^–benzene complexes [1, 6, 8, 26]. The outlined stability trends of Δ*E*_f_ are also preserved for the enthalpies (Δ*H*_f_) and Gibbs free energies (Δ*G*_f_, Table 2). Although a strong destabilizing entropic (*T*Δ*S*) contribution is also observed (∼6–7 kcal/mol), all of the complexes are stable according to their Δ*G*_f_ values at both the MP2 and PBE levels (Table 2).

**Fig. 4:**
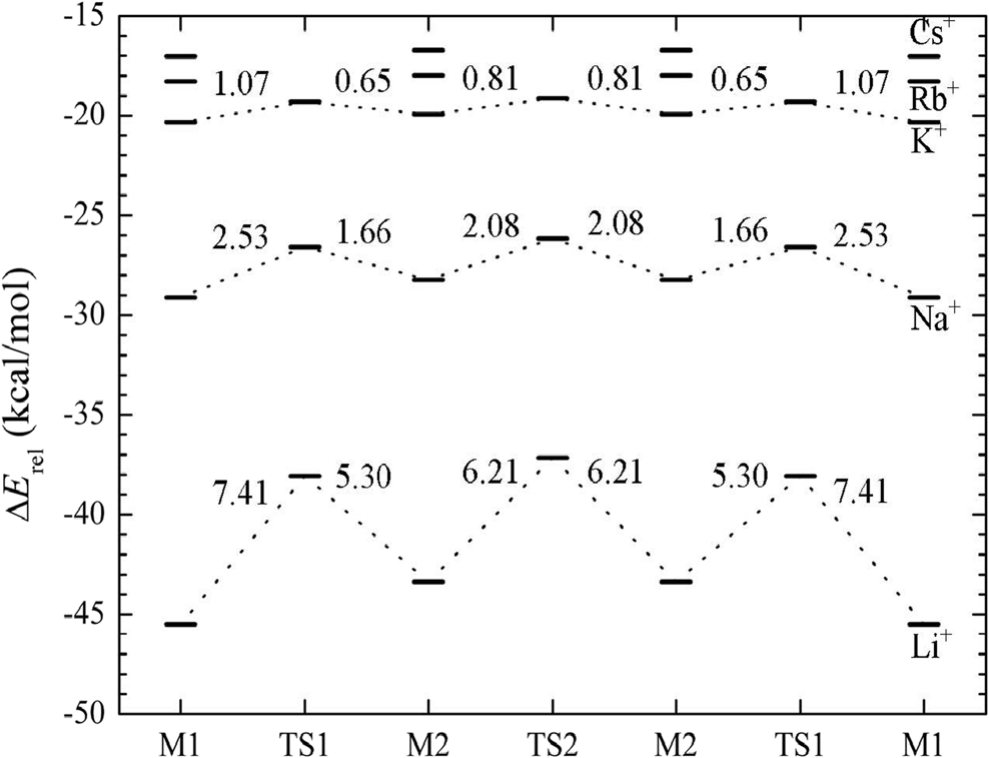
Energy profiles and transition barriers (kcal/mol) for alkali metal cation–pyrene complexes

As also done for the H^+^–pyrene complexes, transition states (TS1, TS2) connecting the energy minima (M1 and M2, Fig. 3) and the corresponding transition energy barriers were calculated for the alkali metal cation–pyrene complexes. The energy profiles and barrier heights (kcal/mol) calculated at the PBE level are presented in Fig. 4. In parallel with a decrease in the difference between the stabilities of the two minima M1 and M2 with increasing cation size, a decrease in transition barrier height is also observed. The smaller alkali metal cations Li^+^ and Na^+^ both have non-negligible transition barriers. For the Li^+^ complex, the transition barrier from M1 to M2 amounts to 7.4 kcal/mol. In the opposite direction (M2 to M1), the barrier drops to 5.3 kcal/mol. Both values agree well with those previously calculated at the B3LYP/6311+G(3df,2p) level: 6.8 and 4.5 kcal/mol, respectively [31]. The barrier to the transition between the inner rings (M2 to M2), 6.2 kcal/mol (Fig. 3), is between the values for the forward (M1 to M2) and backward (M2 to M1) transitions.

The K^+^–pyrene complex represents an intermediate case between the smaller and bigger cations. Its barrier is around 1 kcal/mol for the transition from M1 to M2 (TS1), and it is even lower for the M2 to M2 transition (TS2) (Fig. 4). Since the transition barrier heights for the big alkali metal cations Rb^+^ and Cs^+^ are expected to be even lower, the respective transition states were not calculated explicitly. Thus, for Rb^+^ and Cs^+^, all possible transitions are expected to be virtually barrierless, indicating that these large alkali metal cations are highly mobile on pyrene (and generally on any large π-system, such as graphene). This conclusion was drawn purely on the basis of energetic considerations. The mobilities of these cations are expected to be even more enhanced when temperature effects are taken into account.

### Influence of the microhydration shell

In order to investigate the influence of the microhydration shell on the adsorption of M^+^ to pyrene and the competition between the M^+^–pyrene and M^+^–water interactions, potential energy curves as a function of the M^+^···pyrene perpendicular distance (*d*(M^+^···pyrene)) have been generated for the isolated Li^+^/K^+^ ions and the corresponding microhydrated complexes Li^+^(H_2_O)_4_ and K^+^ (H_2_O)_8_, respectively. During the optimization process, the pyrene molecule and the cation were fixed and the water molecules were relaxed at each point on the potential energy curve. For both Li^+^ and K^+^, representing small and big alkali metal cations, respectively, the position at the M2 site (Fig. 3) was considered. The interaction at the M1 site was expected to give very similar trends, and was therefore not taken into account.

**Fig. 5:**
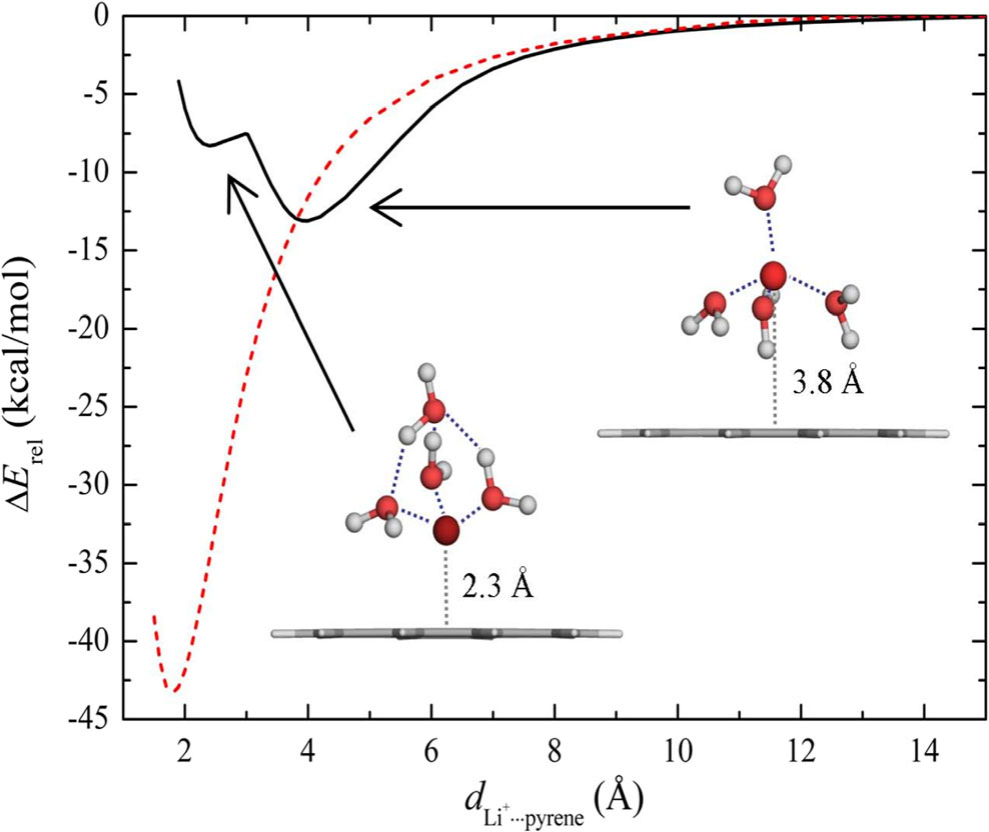
Potential energy curves for isolated Li^+^–pyrene (*dashed line*) and microhydrated Li^+^(H_2_O)_4_–pyrene (*solid line*) complexes

The potential energy (PE) curves for the isolated and microhydrated Li^+^ complexes with pyrene are presented in Fig. 5. The PE curve of the isolated Li^+^··pyrene complex has only one minimum (M2) at a distance of ∼1.8 Å with an energy of −45.5 kcal/mol (PBE/def2-TZVPP level, Table 2) with respect to the individual components at infinite separation. On the other hand, for the microhydrated Li^+^ (H_2_O)_4_–pyrene complex, two minima—both significantly higher in energy than for the nonhydrated case—are found at distances of *d* = 2.3 and 4.0 Å, respectively. Evidently, hydration strongly destabilizes the Li^+^–pyrene complex. The local minimum of the PE curve for the microhydrated Li^+^ observed at 2.3 Å corresponds to the formation of an inner complex where Li^+^ is in direct contact with the aromatic system, as in the case of the bare Li^+^–pyrene complex. However, the effect of hydration by four water molecules shifts the Li^+^ ∼0.5 Å further away from the pyrene plane. The other (global) minimum at ∼3.8 Å corresponds to an outer complex where Li^+^ is hydrated by four water molecules in a tetrahedral coordination (Fig. 5). Three water molecules from the coordination shell have hydrogen atoms oriented towards the pyrene plane; these H atoms form weak hydrogen bonds (lengths ∼2.5 Å) with the π-system. The outer complex is ∼5 kcal/mol more stable than the inner one. This indicates that Li^+^ favors cation–water over cation–π interactions.

**Fig. 6:**
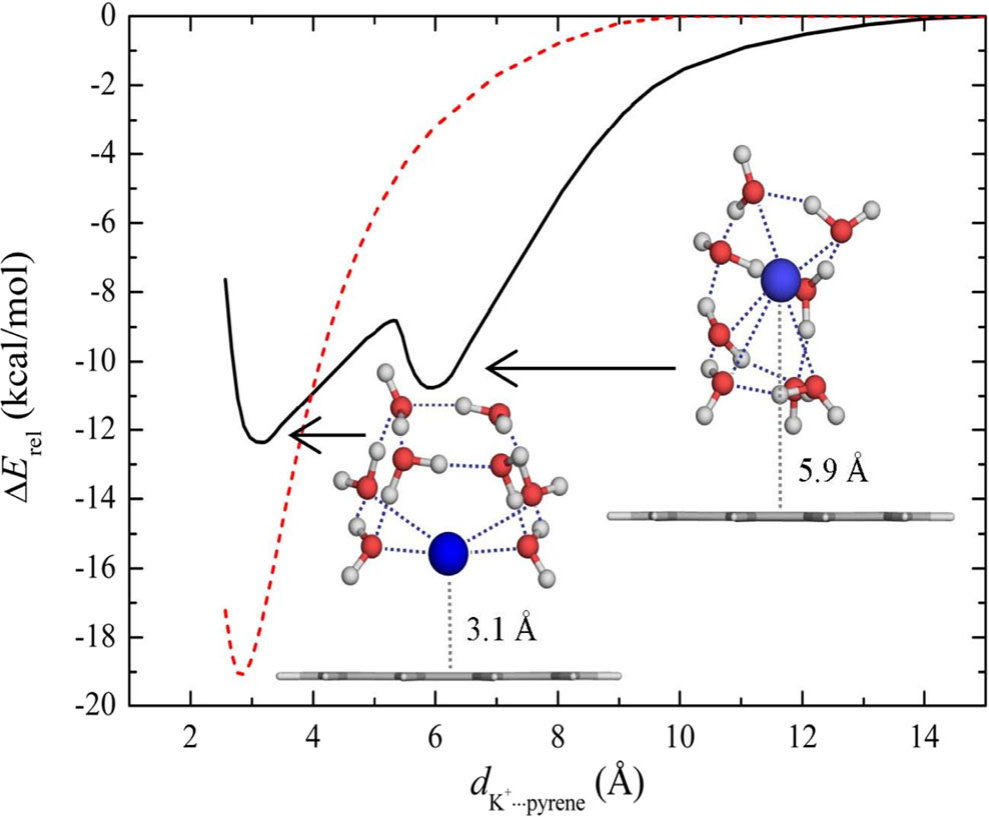
Potential energy curves for isolated K^+^–pyrene (*dashed line*) and microhydrated K^+^(H_2_O)_8_–pyrene (*solid line*) complexes

For the isolated K^+^–pyrene complex (Fig. 6), a similar PE curve to that reported for the respective isolated Li^+^–pyrene complex (Fig. 5) is obtained. The minimum found at a distance of ∼2.8 Å corresponds to the M2 site. The PE curve of the microhydrated K^+^(H_2_O)_8_–pyrene complex has two minima, just like the Li^+^(H_2_O)_4_–pyrene complex. The minima reflect the formation of inner- and outer-sphere K^+^(H_2_O)_8_–pyrene complexes, respectively. However, the relative energetic stability of the inner compared to the outer microhydrated K^+^(H_2_O)_8_–pyrene complex is very different from the relative energetic stability of the inner compared to the outer microhydrated Li^+^ complex. The cation in the inner microhydrated K^+^(H_2_O)_8_–pyrene complex is located ∼3.1 Å from the pyrene plane (about 0.3 Å further than in the bare complex). This complex is more stable than the outer complex (with a K^+^···pyrene distance of 5.9 Å) by about 1.5 kcal/mol. Thus, in contrast to Li^+^, K^+^ favors cation–π over cation–water interactions. Moreover, the complex is also stabilized by hydrogen bonds of H_2_O···π type between two water molecules and the pyrene (Fig. 6), with H···pyrene distances of about 2.5 Å. The microhydration shell of the inner complex is arranged in a hemisphere-like configuration around the K^+^ cation, with only four of the eight water molecules coordinated directly to the cation (the K^+^···O distances are about 2.8 Å). The remaining four water molecules form a square on top of the complex with K^+^···O distances of 3.9–4.1 Å. Overall, in the hemisphere-like water cluster, strong hydrogen bonds are formed among the water molecules with H···O distances of ∼1.4–1.7 Å. The observed differences between the microhydrated pyrene–Li^+^ and pyrene–K^+^ clusters agree well with the conclusions drawn from a previous experimental investigation by Cabarcos et al. [29] of Na^+^/K^+^–(benzene)_*m*_(H_2_O)_*n*_ clusters, where similar differences between Na^+^ and K^+^ complexes were observed. In the K^+^ clusters, the cation was stabilized by benzene molecules and water molecules were displaced further away, but in the Na^+^ clusters, the water molecules remained (stabilized) in the first coordination shell. Similar findings were reported by Meng et al. [35] from their ab initio molecular dynamics study of microhydrated Na^+^ and K^+^ cations on a graphite surface. These different complexation patterns of hydrated alkali cations with aromatic systems can be attributed to ionic size selectivity effects [29], in which the interplay between several factors such as cation–π interactions, cation–water complexation, and the hydrogen bonding of water to the π-system plays an important role.

**Fig. 7a–b:**
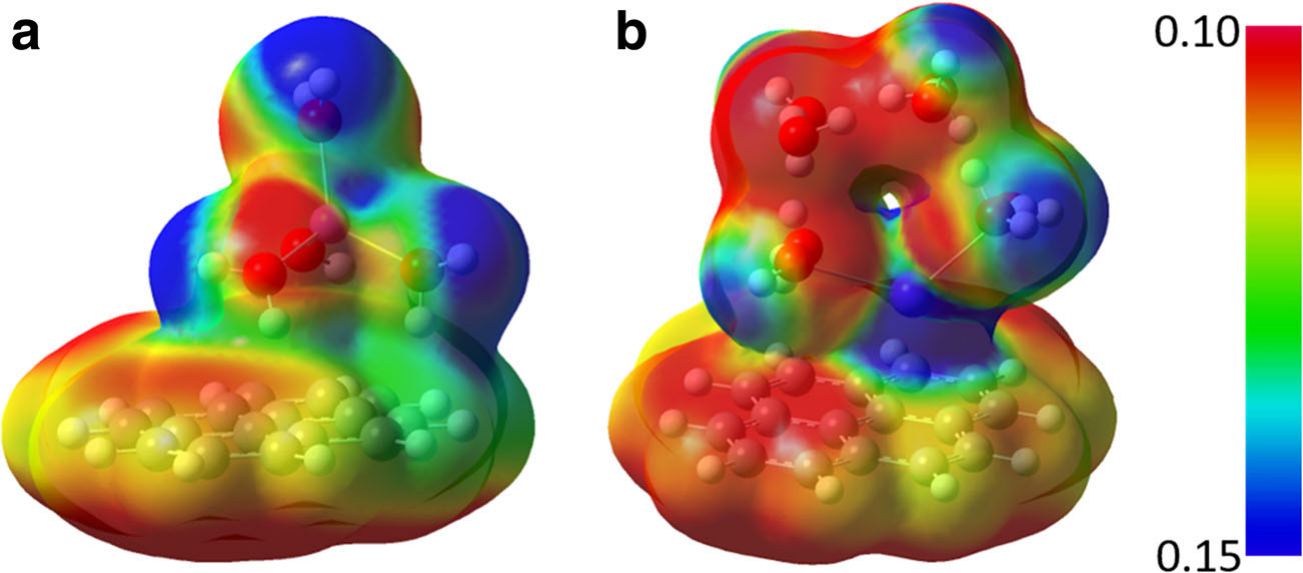
Calculated electrostatic potential (in hartrees) mapped onto the 0.001 *e*/bohr^3^ electron density isosurfaces for Li^+^(H_2_O)_4_–pyrene (**a**) and K^+^(H_2_O)_8_–pyrene (**b**). The scale for the electrostatic potential is positive because the total charge on both systems is +1

To get a better understanding of the difference between the interactions of microhydrated Li^+^ and K^+^ cations with pyrene, we compared the electrostatic potential surfaces of both systems (Fig. 7). The electrostatic potential is a useful physical property, and calculated values of it (e.g., on a molecular surface) can indicate which parts of a molecular system are important for nonbonding interactions [54]. The calculated electrostatic potential was mapped onto the 0.001 *e*/bohr^3^ electron density isosurfaces for the structures at the global minima of the potential energy curves, i.e., the outer complex for the Li^+^ case and the inner complex for K^+^ (Figs. 5 and 6). A key factor in the configuration of the microhydrated cations on the pyrene surface is the cation radius. The small Li^+^ cation polarizes water molecules more than K^+^ does, and is screened by them from the pyrene π-system. The polarized water molecules in turn polarize a large part of the aromatic system of pyrene. On the other hand, the much larger K^+^ cation—which is in direct contact with pyrene—directly polarizes the π-system more than the water molecules from its incomplete coordination shell. Moreover, the water molecules interact more weakly with pyrene than the water molecules in the Li^+^(H_2_O)_4_–pyrene system do.

## Conclusions

Cation–π interactions have been investigated for H^+^–pyrene and alkali metal cation M^+^–pyrene complexes (M = Li, Na, K, Rb, and Cs) by means of MP2 and DFT/PBE calculations. In H^+^–pyrene, the proton forms chemical bonds with different carbon atoms of pyrene. A detailed stability analysis of all possible protonation sites shows that the outer ring carbon atoms that are bonded to hydrogen atoms are preferred for protonation over the central carbon atoms. This is in accord with resonance effects based on valence bond theory. Analysis of the stabilities of the different protonation sites in pyrene showed that the peripheral carbon atoms (bonded to hydrogen atoms) are much more stable than the inner carbon atoms. The barriers between the stable structures protonated at the peripheral carbon atoms are relatively high (greater than ∼7 kcal/mol). Only the transition barrier from the least stable site (II) to the most stable one (III, α-carbon) is low (2.3 kcal/mol).

For the M^+^–pyrene complexes, two energy minima (M1 and M2) were found in which the cation is located above the outer and inner pyrene rings, respectively. For the smaller cations (Li^+^ and Na^+^), M1 is more stable than M2 by about 2 kcal/mol for the Li^+^–pyrene complex. For the bigger cations (K^+^, Rb^+^, and Cs^+^), the M1 and M2 structures present almost (within a few tenths of a kcal/mol) the same stability. Both the binding energies and the transition energy barriers between the outer (M1) and inner (M2) rings decrease with increasing cation size from Li^+^ to Cs^+^. For K^+^, which represents an intermediate case between small and big alkali metal cations, the transition barrier heights are about 1 kcal/mol. The transition barriers for the bigger Rb^+^ and Cs^+^ cations are estimated to be lower than 1 kcal/mol, indicating practically barrierless transitions and high mobilities of these cations on the aromatic surface.

To investigate the influence of the water molecules that coordinate M^+^ on its adsorption to pyrene, potential energy curves for the isolated Li^+^–pyrene and K^+^–pyrene and microhydrated Li^+^(H_2_O)_4_–pyrene and K^+^(H_2_O)_8_–pyrene complexes were calculated. Li^+^ and K^+^ were selected as representatives of small and big alkali metal cations, respectively. While only one minimum was found for the isolated complexes, the microhydrated complexes exhibited two minima, corresponding to an inner- and an outer-shell complex, respectively. For Li^+^, the outer complex was found to be more stable (by ∼5 kcal/mol) than the inner one, indicating that Li^+^ favors cation–water over cation–π interactions. The relatively strong tetrahedral hydration shell of Li^+^ remains intact upon adsorption to the aromatic system. Although two minima reflecting the inner and outer complexes were also found for the microhydrated K^+^(H_2_O)_8_–pyrene complex, the outer complex is more stable than the inner one by about 1.5 kcal/mol, in contrast to the situation for Li^+^. Consequently, K^+^ favors cation–π over cation–water interactions. The loose hydration shell allows some water molecules to be displaced quite easily by the aromatic system, leading to partial dehydration of K^+^. The calculated electrostatic potential map showed that the cation radius is a key influence on the polarization of the water molecules and the aromatic system. The results demonstrated how ion size selectivity leads to the formation of different types of complexes (inner sphere and outer sphere, respectively) between microhydrated cations and aromatic systems. The formation mechanism of the complex depends on the balance between cation–π interactions, cation–water complexation, and the hydrogen bonding of water to the π-system.
